# Supplementary material for: On distinguishing between canonical tRNA genes and tRNA gene fragments in prokaryotes
Source: RNA Biol. 2023 Feb 2;20(1):48–58. doi: 10.1080/15476286.2023.2172370 (PMC9897764; doi:10.1080/15476286.2023.2172370)
Supplement: Supplemental Material [file KRNB_A_2172370_SM4287.zip › supplementary_information_thermococcus.pdf]

SUPPLEMENTARY INFORMATION

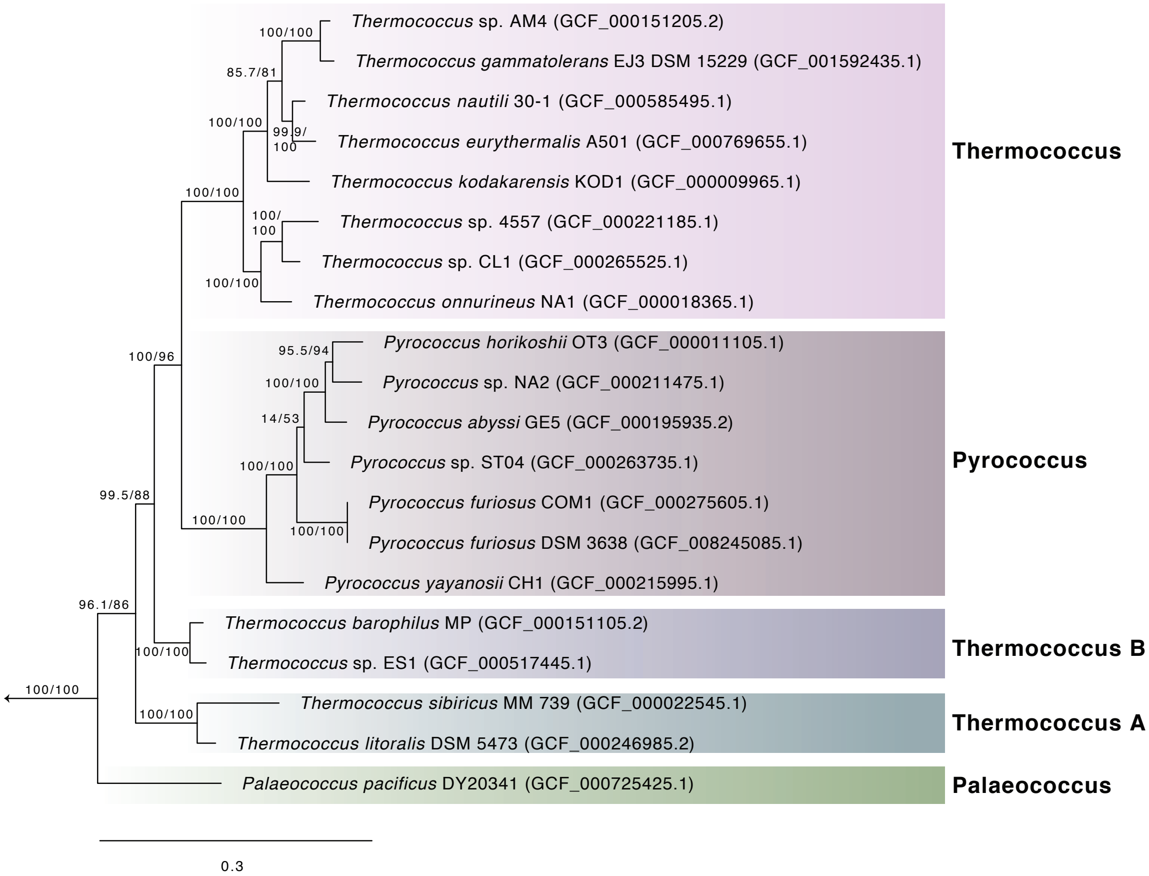

Supplementary Figure S1. Phylogenetic tree highlighting the evolutionary relationships of the 20 Thermococcaceae genomes listed in GtRNAdb. The complete sequences of all genomes were obtained (see Supplementary Tables S1 and S5). Alignments were generated using 12,403 amino acid positions across 43 shared marker proteins (see Supplementary Table S5). A maximum-likelihood phylogenetic tree was inferred with the LG+C60+F+R model with a SH-like approximate likelihood tests (left) and ultrafast bootstrap approximation (right), each run with 1000 replicates. Three genomes – *Methanococcus vannielii SB*, *Methanococcus maripaludis*, and *Methanococcus aeolicus* – were used to root the tree. For further details see Materials and Methods.

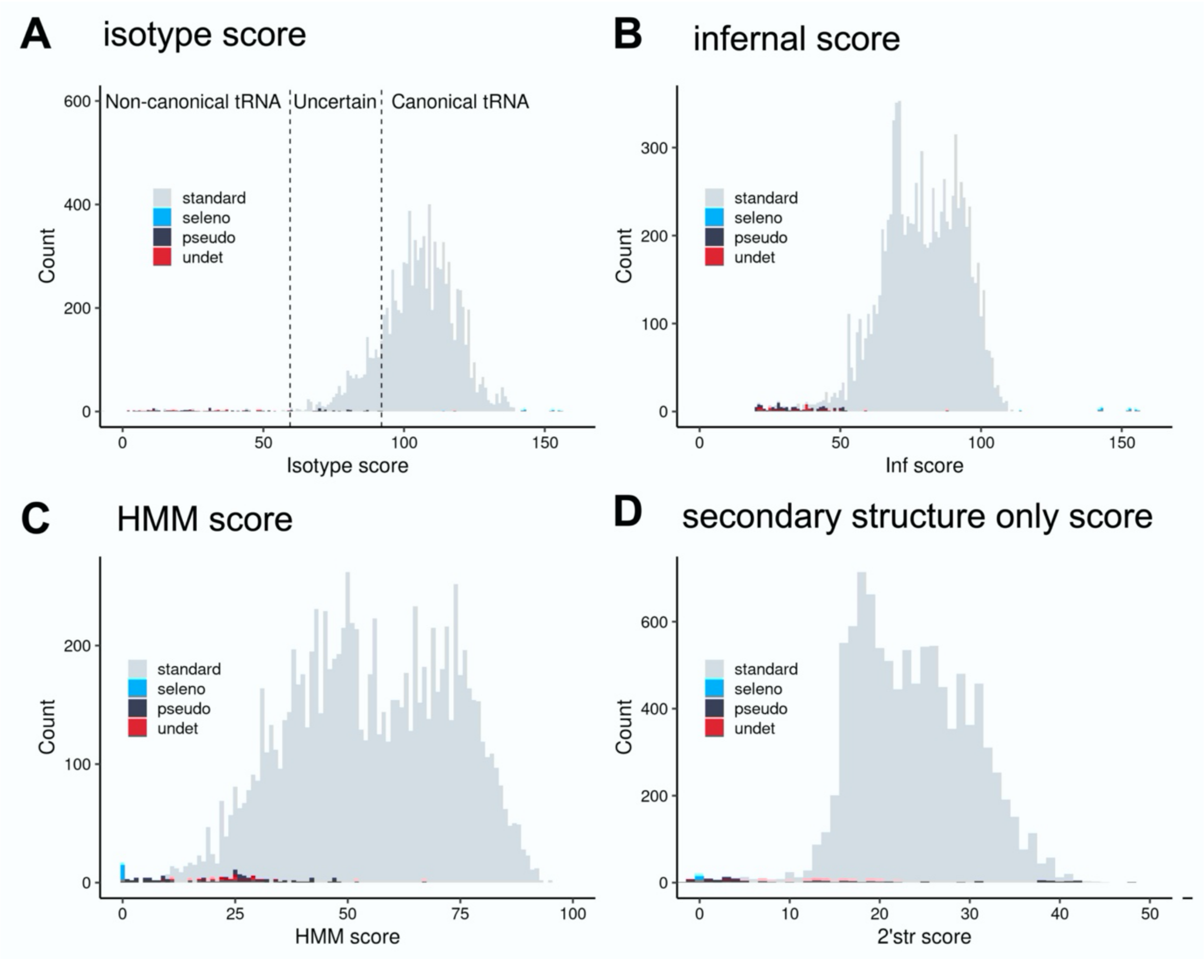

Supplementary Figure S2. tRNAscan-SE isotype score can be used as an indicator of whether a putative archaeal tRNA gene is canonical or non-canonical. Frequencies of locally run tRNAscan-SE (version 2.0.6)-derived scores for ~10,000 putative tRNA genes in 210 archaeal genomes. (**A**) Isotype score (also presented in Figure 5B). (**B**) Infernal score, (**C**) HMM score, and (**D**) Secondary structure-only score. See Chan and Lowe 2021 (doi:10.1093/nar/gkab688) and references therein for a detailed description of score calculations.

Supplementary Tables S1-S7 are excel files, and are all accessible through the following link (legends listed below):

https://www.biorxiv.org/content/10.1101/2022.07.05.498093v3.supplementary-material

Supplementary Table S1: Genomic characteristics of the 20 Thermococcaceae organisms listed on GtRNAdb.

Supplementary Table S2: Evidence for partial tRNA sequences resulting from the integration of genetic elements in the 20 *Thermococcaceae* genomes. Genomic details of seven partial tRNA fragments resulting from the integration of prophages (*i.e.*, erroneously identified as canonical tRNA genes; category 1b in Table 1) in *Thermococcaceae.*

Supplementary Table S3: Evidence for partial tRNA sequences resulting from the integration of genetic elements across Archaea and Bacteria. Genomic details of 17 partial tRNA fragments resulting from the integration of prophages in Archaea and Bacteria.

Supplementary Table S4: Details of ribosomal RNA (*rrn*) operons and associated tRNA genes in the 20 *Thermococcaceae* genomes. Only one genome differs from the norm of a single *rrn* operon and Ala-TGC gene: *Pa. pacificus* DY20341 carries two *rrn* operons and thus two Ala-TGC genes (*i.e.*, a real deviation; category 3).

Supplementary Table S5: Details of markers for drawing of the Thermococcaceae phylogenetic tree.

Supplementary Table S6: tRNA gene predictions, annotations, and scores for 20 Thermococcaceae organisms. Tab 1: downloaded from GtRNAdb. Tab 2: predicted using locally run tRNAscan-SE (version 2.0.6, as for the GtRNAdb entries in Tab 1).

Supplementary Table S7: tRNA gene predictions, annotations, and scores for 210 Archaea. Tab 1: downloaded from GtRNAdb. Tab 2: predicted using locally run tRNAscan-SE (version 2.0.6, as for the GtRNAdb entries in Tab 1).

Supplementary Text S1. Primary and predicted secondary structures of putative tRNA genes of interest. Primary sequences were obtained from GtRNAdb. Secondary structures and listed scores were predicted using the web based tRNAscan-SE 2.0 (sequence source: archaeal; search mode: default).

1 Ser-CGA (+) in T. gammatolerans EJ3

```
>T_gammatolerans_Ser-CGA-2-1
GGAGTAGCCTTCTAAGCCGGAGGtCGCGGGTTCGAATCCCGCCGGGCCCGCCA
```

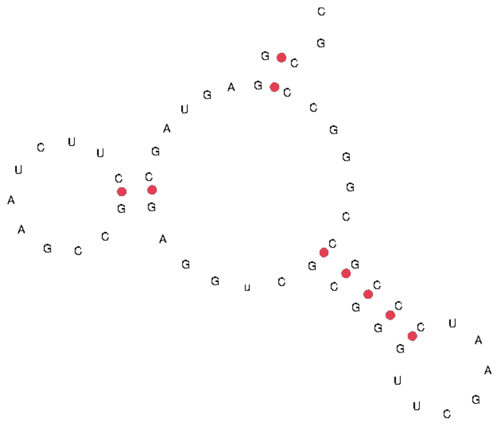

Infernal Score:
22.6

Isotype Score:
10.4

2 Leu-CAA (+) in T. nautili 30-1

```
>T_nautili_Leu-CAA-2-1
GGAGCGGTAGCGGTaACCCCAGCTTTCAAttctcctcgagtcttattgcaacttcctccttctgcaggccgatac
cgcacccccttatcctttcaattctcctcgagtcttattgcaacacgctcgaacagcacataaaagcgatagctga
cgcctttcaattctcctcgagtcttattgcAACGTTGGtCGGGGGTTCAAATCCCCTCCccgGCTCCA
```

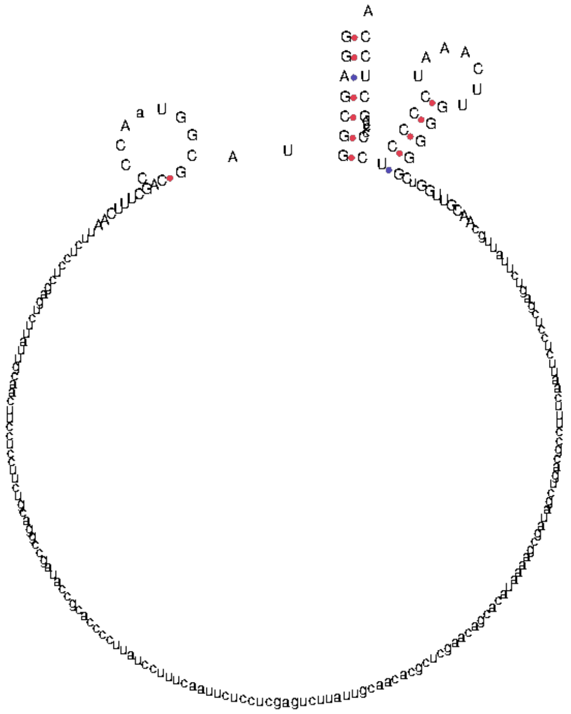

Infernal Score:
22.2

Isotype Score:
2.3

3 Arg-TCT (+) in T. kodakarensis KOD1

>T_kodakarensis_Arg-TCT-2-1

GGCCCTTTCAGCAGCccacaattcattaAGGGAACGGCCTTCTAAGCCGGAGGtCGCGGGTTCGAATCCCGCCGG
GCCCGCCA

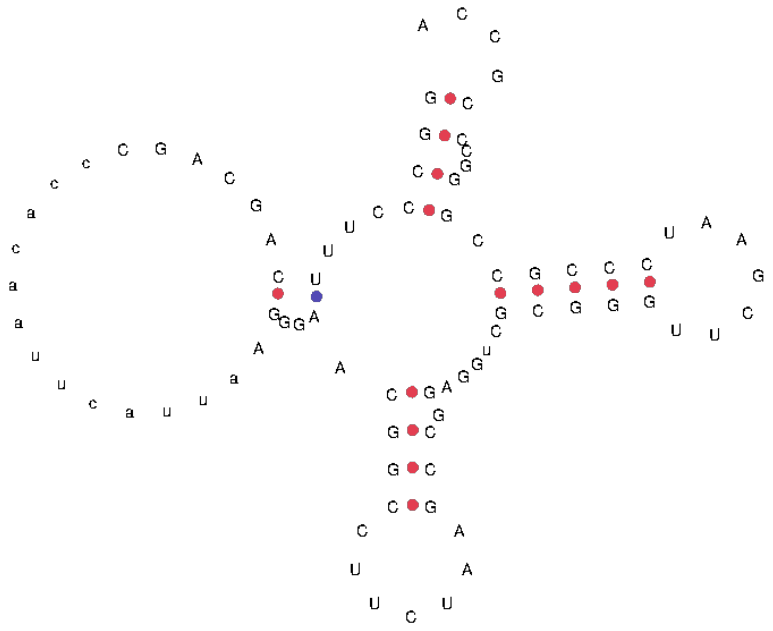

Infernal Score:
27.1

Isotype Score:
21.4

4 Val-CAC (+) in T. kodakarensis KOD1

>T_kodakarensis_Val-CAC-2-1
GGTGTCCcAACAGTCGTTtAGACTGCCCTCACACGGCGGAGGtCCGGGGTTCGAATCCCCGCGGGCCCACCA

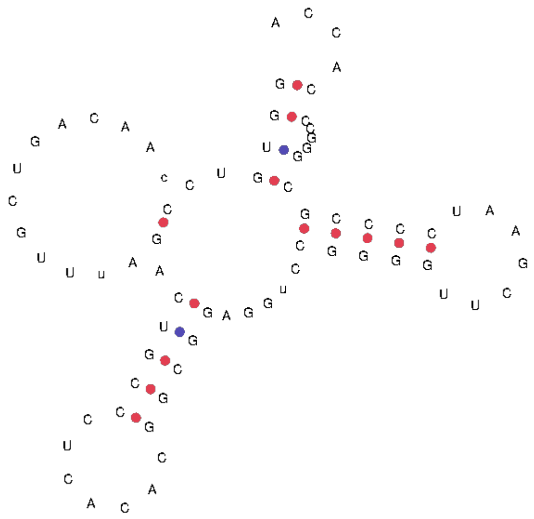

Infernal Score:
45.3

Isotype Score:
83.4

5 Gln-TTG (+) in P. yayanosii CH1

>P_yayanosii_Gln-TTG-2-1
AGCCCTGTAagaCCTTCATTtGGCgaatAAAGGcttttccgACGGGCTTTGGATCCCGCGACTCGGGTTCGAATC
CCGGCGGGGCTACCA

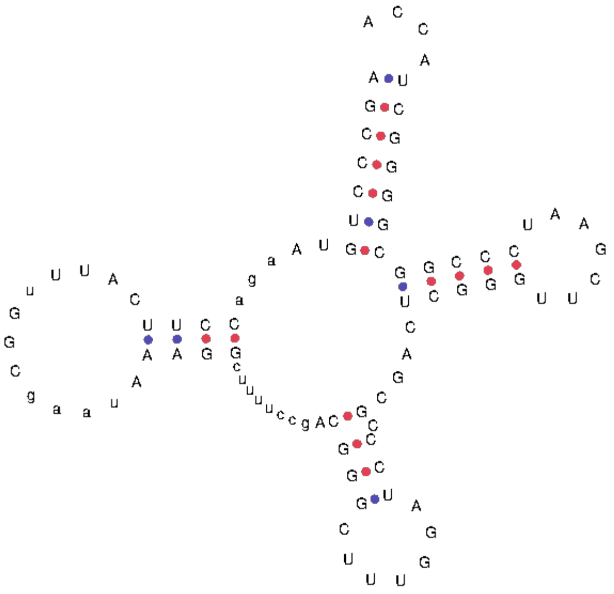

Infernal Score:
26.5

Isotype Score:
13.4

6 Ser-CGA (+) in T. sp ES1

>T_ES1_Ser-CGA-2-1
GGCTGTTTTGCGGTttTGCTGaCCCTTACGAGGCGGAGGtCCGGGGTTCGAATCCCCGCGGGCCCACCA

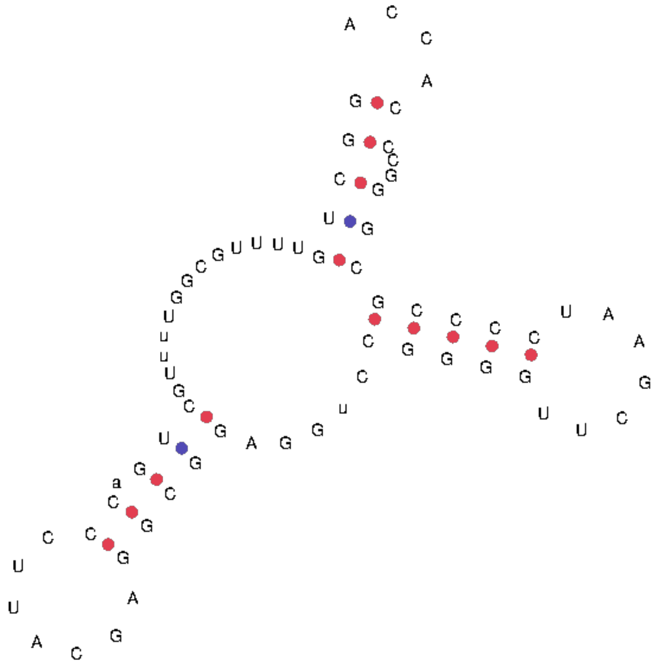

Infernal Score:
33.6

Isotype Score:
30.9

7 Und-NNN (+) in T. sp ES1

>T_ES1_Und-NNN-1-1
GGGTTTGTAGAGGGgAACAAGCCTTCTGctgggtctattagctcaatagccgatgacaaactctctaaattctcc
aaaaaccgctttcgAGCCCGCGcCCCGGGTTCAAATCCCGGCCGGGGCACCA

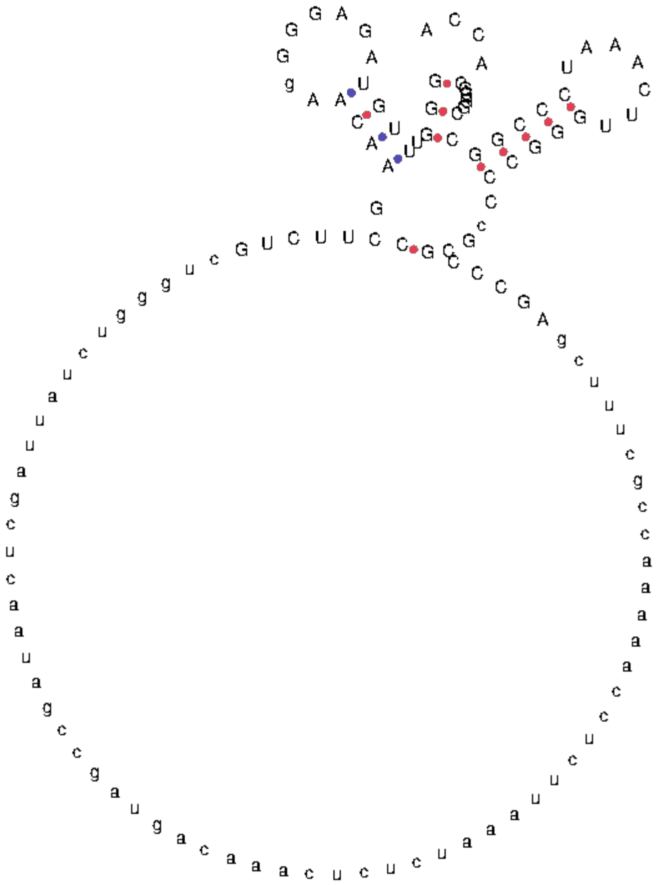

Infernal Score:
25.5

Isotype Score:
16.8

8 Leu-CAA (+) in T. litoralis DSM 5473

>T_litoralis_Leu-CAA-2-1
GGGTCGTTTTTtGGATtTAATagtttagacggctgtgGATCCCCTAGCCCGGGTTCAAATCCCGGCCCCGGCCCC
A

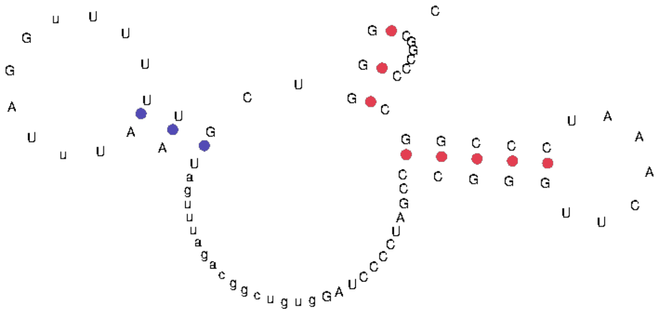

Infernal Score:
21.0

Isotype Score:
6.3

9 & 10 Leu-NAG (+) and Leu-CAG (-) in T. litoralis DSM 5473

```
>T_litoralis_Leu-NAG-1-1 (Leu-CAG)
GCGGGGGTTGCCGAGCCtGGTcaAAGGCGCGGGATTNAGGGTCCCGTCCCGTAGGGGTtCCGGGGTTCAAATCCC
CGCCCCCGCACCA
```

Leu-NAG

Infernal Score:
93.6

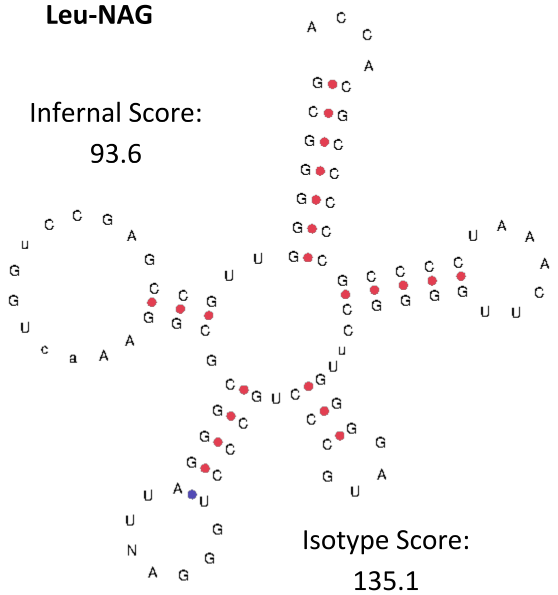

Isotype Score:
135.1

Leu-CAG
(suggested)

Infernal Score:
94.2

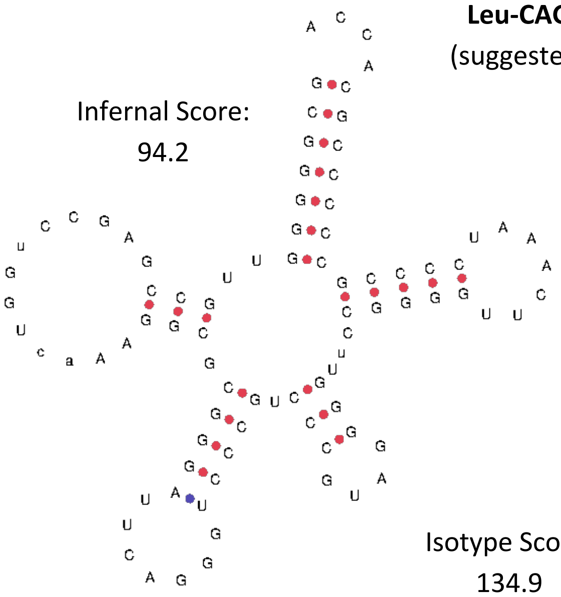

Isotype Score:
134.9

11 & 12 Pro-NGG (+) and Pro-GGG (-) in T. litoralis DSM 5473

```
>T_litoralis_Pro-NGG-1-1 (Pro-GGG)
GGGGCCGTGGGGTAGCTtGGTctATCCTNCCGGCTTNGGGNGCCGGAGACCCGGGTTCAAATCCCGGCGGCCCCA
CCA
```

Pro-NGG

Infernal Score:
91.4

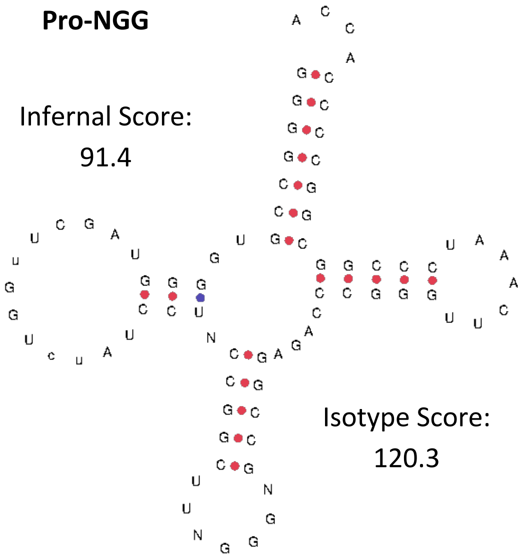

Isotype Score:
120.3

Pro-GGG
(suggested)

Infernal Score:
91.4

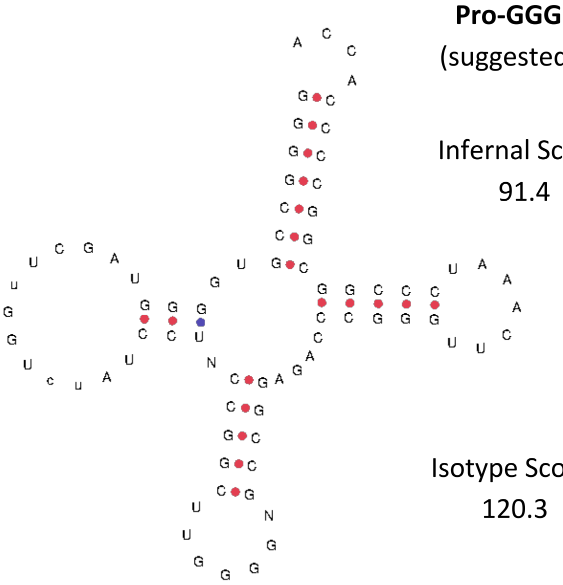

Isotype Score:
120.3

13 & 14 Und-NTG (+) and Gln-CTG (-) in T. litoralis DSM 5473

```
>T_litoralis_Und-NTG-1-1 (Gln-CTG)
AGCCCCGTGGTGTAGCGGCcaAGCATGCGGGACTNTGGATCCCGCGACCGGGGTTCGAATCCCCGCGGGGCTACC
A
```

Und-NTG

Infernal Score:
88.4

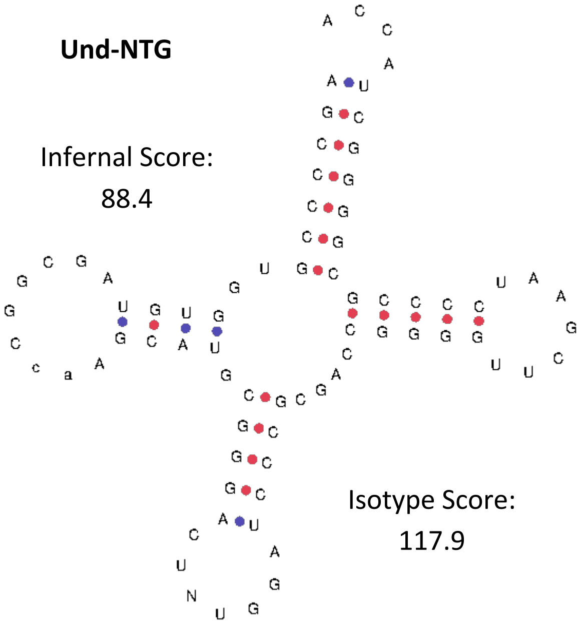

Isotype Score:
117.9

Gln-CTG
(suggested)

Infernal Score:
89.1

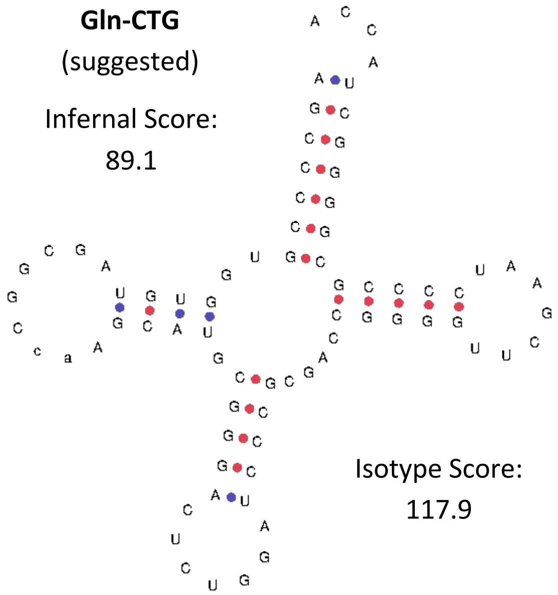

Isotype Score:
117.9

15 Ala-TGC (+) in Pa. pacificus DY20341

```
>Pa_pacificus_Ala-TGC-1-2
GGGCCGGTAGCTCAGCCtGGGAGAGCGCCGGCTTTGCAAGCCGGAGGcCCCGGGTTCAAATCCCGGCCGGTCCAC
CA
```

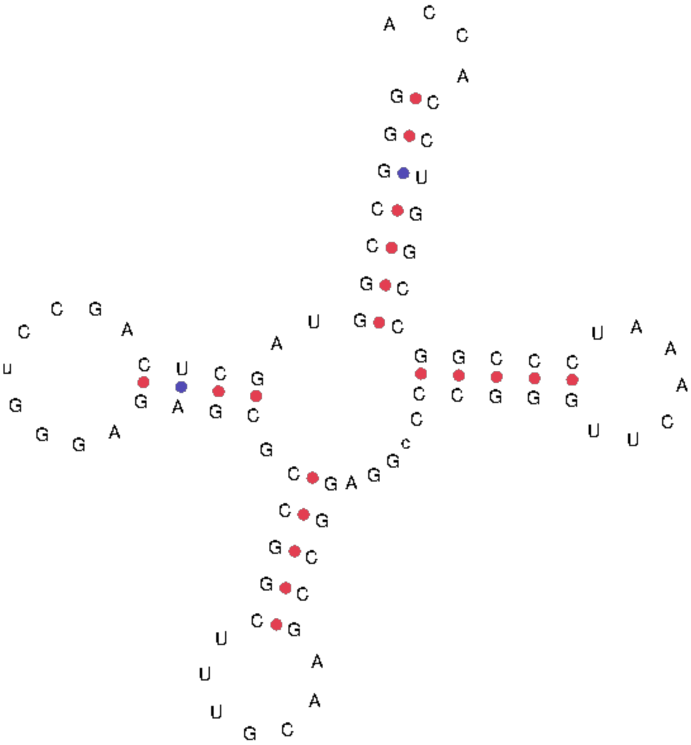

Infernal Score:
102.2

Isotype Score:
122.6

Figure 4: Predictions for the P. furiosus *Arg-GCG tRNA genes*

In Figure 4C-D, the Arg-GCG tRNA gene is from *P. furiosus* DSM3638; it appears to be a true standard tRNA gene with a relatively low tRNA Model Score (85.5). This relatively low score seems to result from a missing 'G' in the later part of the primary sequence, generating a short acceptor stem with an unpaired 'C'. A close relative, *P. furiosus* COM1*,* carries a Arg-GCG containing the missing 'G' base. Hence, we propose this is either an error in the genome sequence of *P. furiosus* DSM3638 genome sequence (or, possibly, a relatively recent mutation).

```
>1_Pfuriosus_DSM3638_Arg-GCG-1-1
GCCCCGGTGGCCTAGCCtGGAtAGGGCGCGAGGCTGCGGACCTCGAGGtCCGGGGTTCAAATCCCCGCCGGGCGC
CA
```

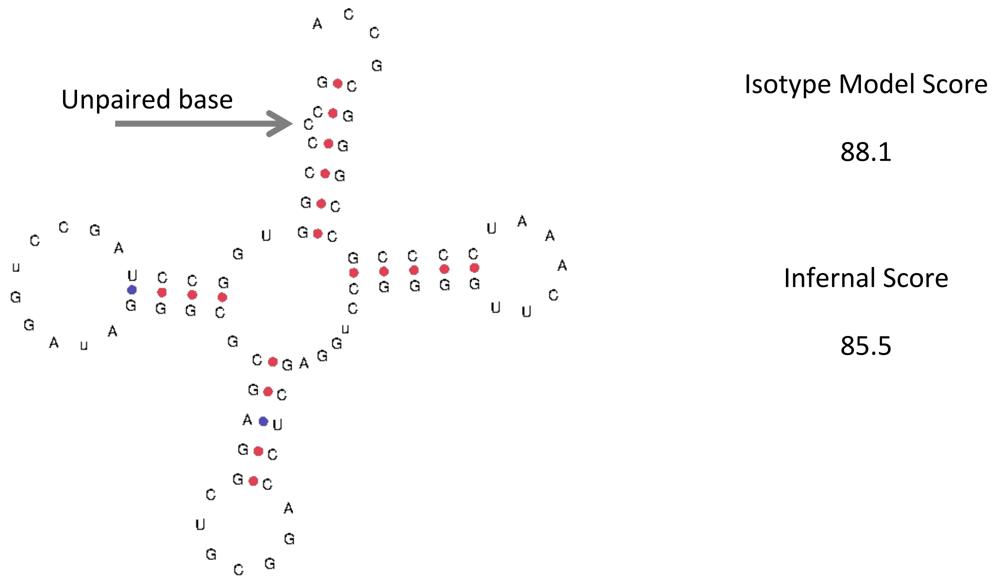


```
>2_Pfuriosus_COM1_Arg-GCG-1-1
GCCCCGGTGGCCTAGCCtGGAtAGGGCGCGAGGCTGCGGACCTCGAGGtCCGGGGTTCAAATCCCCGCCGGGGCG
CCA
```

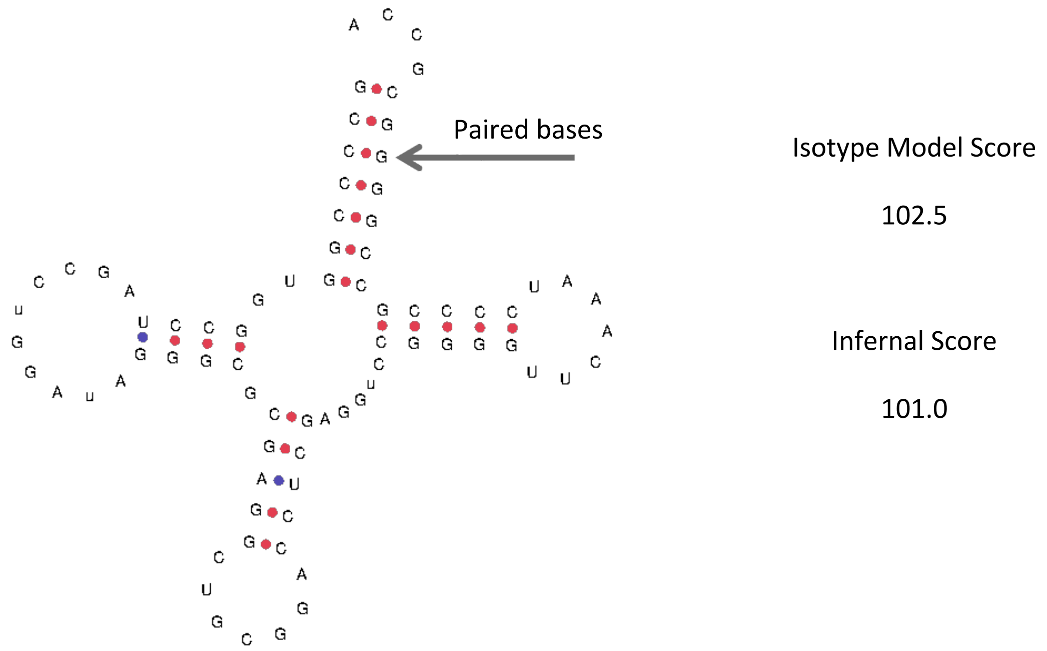

Low-scoring tRNA gene example: Ala-CGC from Thermoplasmatales archaeon *BRNA1*

The GenBank accession for this genome is CP002916.1. We note that it is predicted by tRNAscan-SE to encode a relatively low-scoring (isotype score) tRNA gene that could conceivably encode a functional, canonical tRNA:

tRNA-Ala-CGC-1-1

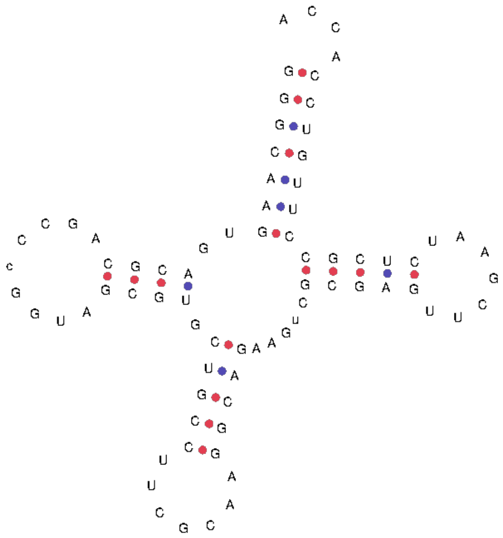

Isotype Model Score

83.7

Infernal Score

79.8
