# Supplementary figures and images for: On distinguishing between canonical tRNA genes and tRNA gene fragments in prokaryotes
Source: RNA Biol. 2023 Feb 2;20(1):48–58. doi: 10.1080/15476286.2023.2172370 (PMC9897764; doi:10.1080/15476286.2023.2172370)

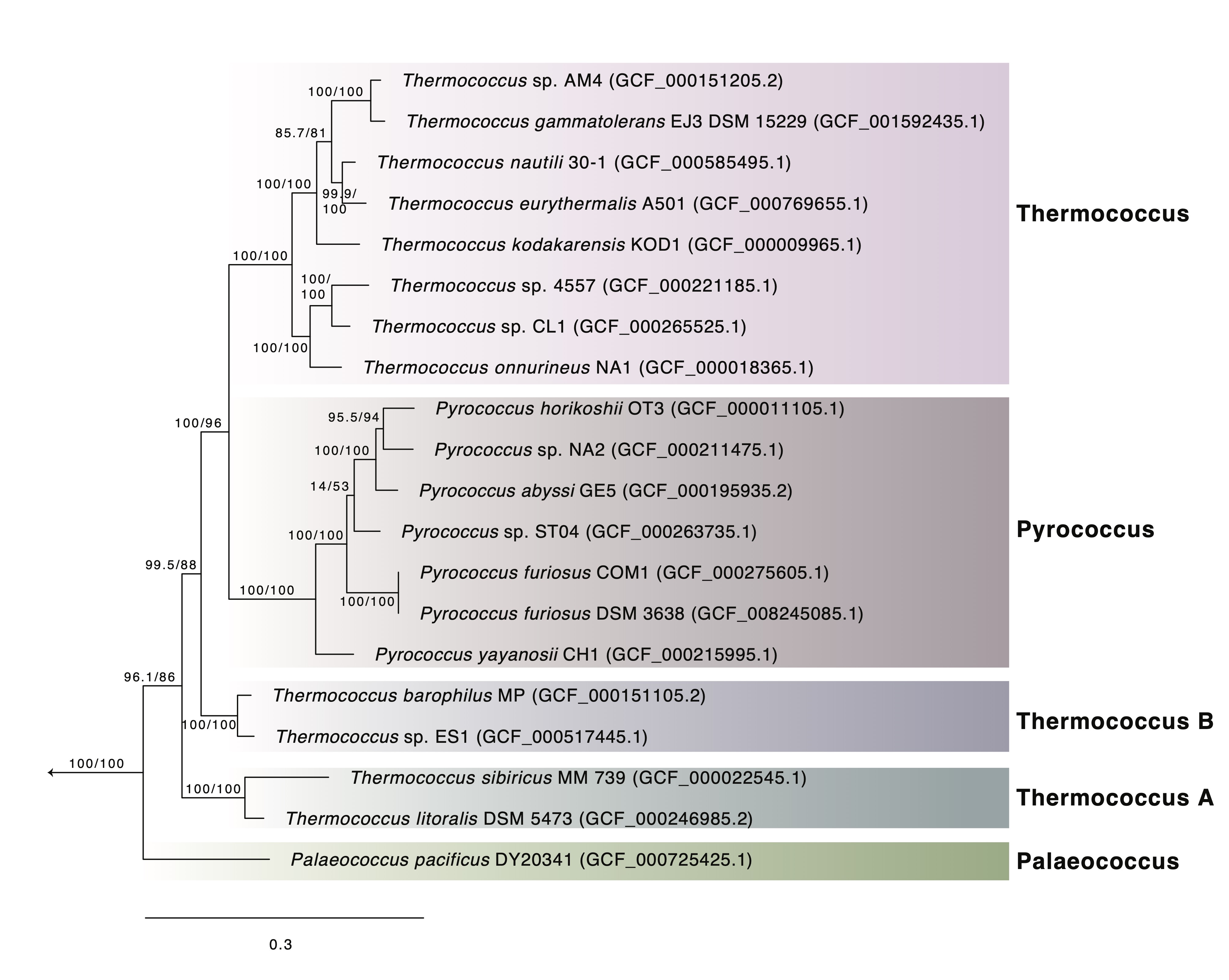

Supplement: Supplemental Material [file KRNB_A_2172370_SM4287.zip › supplementary_figure1.jpg]

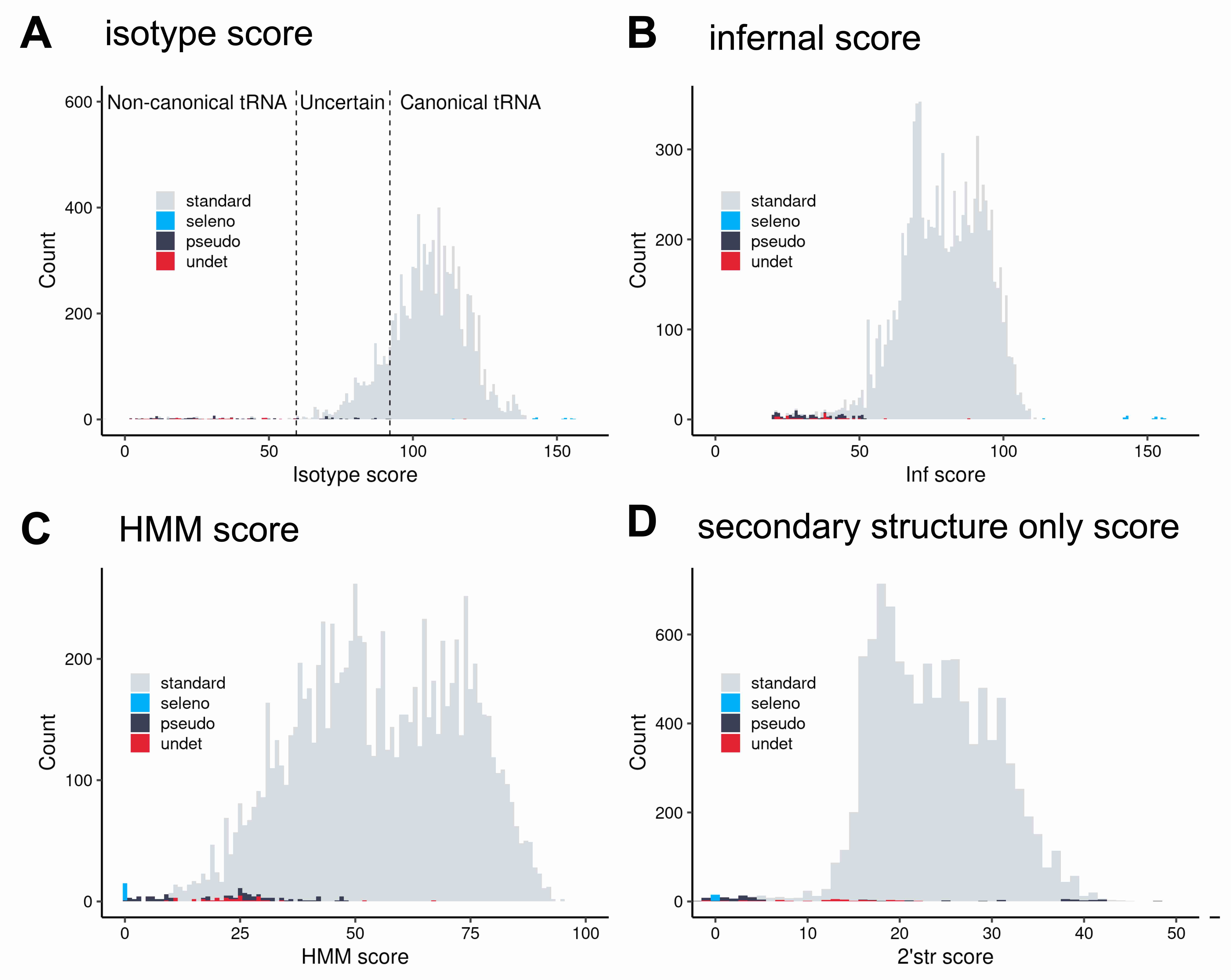

Supplement: Supplemental Material [file KRNB_A_2172370_SM4287.zip › supplementary_figure2.jpg]
